# Supplementary material for: Pyruvate Dehydrogenase Kinase 1 inhibition mediated oxidative phosphorylation enhancement in cartilage promotes osteoarthritis progression
Source: BMC Musculoskelet Disord. 2023 Jul 20;24:597. doi: 10.1186/s12891-023-06585-6 (PMC10357736; doi:10.1186/s12891-023-06585-6)
Supplement: Supplementary file 1 — Additional file 1. [file 12891_2023_6585_MOESM1_ESM.zip › MF.Gsea.1653627316242/GOMF_PHOSPHATASE_REGULATOR_ACTIVITY.html]

Details for gene set GOMF\_PHOSPHATASE\_REGULATOR\_ACTIVITY[GSEA]

|  || Dataset | 55457\_collapsed\_to\_symbols.cls.cls #OA\_versus\_normal.cls.cls #OA\_versus\_normal\_repos |
| Phenotype | cls.cls#OA\_versus\_normal\_repos |
| Upregulated in class | normal |
| GeneSet | GOMF\_PHOSPHATASE\_REGULATOR\_ACTIVITY |
| Enrichment Score (ES) | -0.34552288 |
| Normalized Enrichment Score (NES) | -1.4368335 |
| Nominal p-value | 0.020344287 |
| FDR q-value | 0.23983769 |
| FWER p-Value | 1.0 |
Table: GSEA Results Summary

  

Fig 1: Enrichment plot: GOMF\_PHOSPHATASE\_REGULATOR\_ACTIVITY      
 Profile of the Running ES Score & Positions of GeneSet Members on the Rank Ordered List

  

| SYMBOL | TITLE | RANK IN GENE LIST | RANK METRIC SCORE | RUNNING ES | CORE ENRICHMENT || 1 | STYXL1 | serine/threonine/tyrosine interacting like 1 [Source:HGNC Symbol;Acc:HGNC:18165] | 244 | 0.787 | 0.0132 | No |
| 2 | PTN | pleiotrophin [Source:HGNC Symbol;Acc:HGNC:9630] | 402 | 0.706 | 0.0298 | No |
| 3 | PPP1R8 | protein phosphatase 1 regulatory subunit 8 [Source:HGNC Symbol;Acc:HGNC:9296] | 885 | 0.547 | 0.0146 | No |
| 4 | CALM3 | calmodulin 3 [Source:HGNC Symbol;Acc:HGNC:1449] | 1039 | 0.510 | 0.0235 | No |
| 5 | URI1 | URI1 prefoldin like chaperone [Source:HGNC Symbol;Acc:HGNC:13236] | 1324 | 0.454 | 0.0199 | No |
| 6 | PPP1R7 | protein phosphatase 1 regulatory subunit 7 [Source:HGNC Symbol;Acc:HGNC:9295] | 1430 | 0.439 | 0.0296 | No |
| 7 | PPP2R5D | protein phosphatase 2 regulatory subunit B'delta [Source:HGNC Symbol;Acc:HGNC:9312] | 2137 | 0.346 | -0.0113 | No |
| 8 | ENSA | endosulfine alpha [Source:HGNC Symbol;Acc:HGNC:3360] | 2172 | 0.341 | 0.0000 | No |
| 9 | PPP2R3B | protein phosphatase 2 regulatory subunit B''beta [Source:HGNC Symbol;Acc:HGNC:13417] | 2224 | 0.336 | 0.0098 | No |
| 10 | PPP1R2 | protein phosphatase 1 regulatory inhibitor subunit 2 [Source:HGNC Symbol;Acc:HGNC:9288] | 2277 | 0.329 | 0.0192 | No |
| 11 | PPP2R5A | protein phosphatase 2 regulatory subunit B'alpha [Source:HGNC Symbol;Acc:HGNC:9309] | 2592 | 0.291 | 0.0066 | No |
| 12 | BMPR1A | bone morphogenetic protein receptor type 1A [Source:HGNC Symbol;Acc:HGNC:1076] | 2745 | 0.275 | 0.0061 | No |
| 13 | B3GAT3 | "beta-1,3-glucuronyltransferase 3 [Source:HGNC Symbol;Acc:HGNC:923]" | 2987 | 0.251 | -0.0025 | No |
| 14 | PPP2R3A | protein phosphatase 2 regulatory subunit B''alpha [Source:HGNC Symbol;Acc:HGNC:9307] | 3149 | 0.235 | -0.0054 | No |
| 15 | PPP2R5C | protein phosphatase 2 regulatory subunit B'gamma [Source:HGNC Symbol;Acc:HGNC:9311] | 3205 | 0.230 | -0.0003 | No |
| 16 | PPP1R11 | protein phosphatase 1 regulatory inhibitor subunit 11 [Source:HGNC Symbol;Acc:HGNC:9285] | 3239 | 0.227 | 0.0064 | No |
| 17 | FRS2 | fibroblast growth factor receptor substrate 2 [Source:HGNC Symbol;Acc:HGNC:16971] | 3385 | 0.213 | 0.0038 | No |
| 18 | IGBP1 | immunoglobulin binding protein 1 [Source:HGNC Symbol;Acc:HGNC:5461] | 3413 | 0.210 | 0.0103 | No |
| 19 | MYOZ1 | myozenin 1 [Source:HGNC Symbol;Acc:HGNC:13752] | 3437 | 0.207 | 0.0170 | No |
| 20 | PPP1R2C | PPP1R2C family member C [Source:HGNC Symbol;Acc:HGNC:16324] | 3881 | 0.166 | -0.0108 | No |
| 21 | PPP2R2D | protein phosphatase 2 regulatory subunit Bdelta [Source:HGNC Symbol;Acc:HGNC:23732] | 4152 | 0.142 | -0.0260 | No |
| 22 | PPP1R12B | protein phosphatase 1 regulatory subunit 12B [Source:HGNC Symbol;Acc:HGNC:7619] | 5058 | 0.068 | -0.0938 | No |
| 23 | MGAT5 | "alpha-1,6-mannosylglycoprotein 6-beta-N-acetylglucosaminyltransferase [Source:HGNC Symbol;Acc:HGNC:7049]" | 5086 | 0.066 | -0.0932 | No |
| 24 | EIF2AK2 | eukaryotic translation initiation factor 2 alpha kinase 2 [Source:HGNC Symbol;Acc:HGNC:9437] | 5145 | 0.060 | -0.0953 | No |
| 25 | IGFBP3 | insulin like growth factor binding protein 3 [Source:HGNC Symbol;Acc:HGNC:5472] | 5167 | 0.059 | -0.0945 | No |
| 26 | PPP2R2B | protein phosphatase 2 regulatory subunit Bbeta [Source:HGNC Symbol;Acc:HGNC:9305] | 5292 | 0.048 | -0.1022 | No |
| 27 | RCAN2 | regulator of calcineurin 2 [Source:HGNC Symbol;Acc:HGNC:3041] | 5399 | 0.040 | -0.1089 | No |
| 28 | BMP2K | BMP2 inducible kinase [Source:HGNC Symbol;Acc:HGNC:18041] | 5760 | 0.011 | -0.1365 | No |
| 29 | PPP1R17 | protein phosphatase 1 regulatory subunit 17 [Source:HGNC Symbol;Acc:HGNC:16973] | 5786 | 0.009 | -0.1381 | No |
| 30 | PPP1R14B | protein phosphatase 1 regulatory inhibitor subunit 14B [Source:HGNC Symbol;Acc:HGNC:9057] | 5791 | 0.009 | -0.1380 | No |
| 31 | PPP2R2A | protein phosphatase 2 regulatory subunit Balpha [Source:HGNC Symbol;Acc:HGNC:9304] | 5946 | -0.001 | -0.1500 | No |
| 32 | BMPR1B | bone morphogenetic protein receptor type 1B [Source:HGNC Symbol;Acc:HGNC:1077] | 5959 | -0.002 | -0.1508 | No |
| 33 | PTPA | protein phosphatase 2 phosphatase activator [Source:HGNC Symbol;Acc:HGNC:9308] | 6120 | -0.014 | -0.1627 | No |
| 34 | PPP1R16B | protein phosphatase 1 regulatory subunit 16B [Source:HGNC Symbol;Acc:HGNC:15850] | 6283 | -0.026 | -0.1743 | No |
| 35 | CABIN1 | calcineurin binding protein 1 [Source:HGNC Symbol;Acc:HGNC:24187] | 6477 | -0.040 | -0.1877 | No |
| 36 | RCAN1 | regulator of calcineurin 1 [Source:HGNC Symbol;Acc:HGNC:3040] | 6727 | -0.058 | -0.2048 | No |
| 37 | PPP1R1A | protein phosphatase 1 regulatory inhibitor subunit 1A [Source:HGNC Symbol;Acc:HGNC:9286] | 7040 | -0.083 | -0.2257 | No |
| 38 | MAP2K6 | mitogen-activated protein kinase kinase 6 [Source:HGNC Symbol;Acc:HGNC:6846] | 7246 | -0.099 | -0.2377 | No |
| 39 | PPP1R26 | protein phosphatase 1 regulatory subunit 26 [Source:HGNC Symbol;Acc:HGNC:29089] | 7288 | -0.101 | -0.2367 | No |
| 40 | GNA12 | G protein subunit alpha 12 [Source:HGNC Symbol;Acc:HGNC:4380] | 7294 | -0.102 | -0.2329 | No |
| 41 | PPME1 | protein phosphatase methylesterase 1 [Source:HGNC Symbol;Acc:HGNC:30178] | 7483 | -0.117 | -0.2428 | No |
| 42 | PHACTR4 | phosphatase and actin regulator 4 [Source:HGNC Symbol;Acc:HGNC:25793] | 7674 | -0.133 | -0.2522 | No |
| 43 | PPP4R4 | protein phosphatase 4 regulatory subunit 4 [Source:HGNC Symbol;Acc:HGNC:23788] | 7902 | -0.148 | -0.2639 | No |
| 44 | PPP4R3B | protein phosphatase 4 regulatory subunit 3B [Source:HGNC Symbol;Acc:HGNC:29267] | 8130 | -0.165 | -0.2748 | No |
| 45 | PPP4R1 | protein phosphatase 4 regulatory subunit 1 [Source:HGNC Symbol;Acc:HGNC:9320] | 8712 | -0.210 | -0.3116 | No |
| 46 | GTF2F1 | general transcription factor IIF subunit 1 [Source:HGNC Symbol;Acc:HGNC:4652] | 8910 | -0.225 | -0.3177 | No |
| 47 | ARPP19 | cAMP regulated phosphoprotein 19 [Source:HGNC Symbol;Acc:HGNC:16967] | 8912 | -0.225 | -0.3086 | No |
| 48 | PPP6R2 | protein phosphatase 6 regulatory subunit 2 [Source:HGNC Symbol;Acc:HGNC:19253] | 8962 | -0.229 | -0.3030 | No |
| 49 | LGALS3 | galectin 3 [Source:HGNC Symbol;Acc:HGNC:6563] | 9508 | -0.274 | -0.3343 | Yes |
| 50 | PPP2R5B | protein phosphatase 2 regulatory subunit B'beta [Source:HGNC Symbol;Acc:HGNC:9310] | 9554 | -0.279 | -0.3264 | Yes |
| 51 | ANP32E | acidic nuclear phosphoprotein 32 family member E [Source:HGNC Symbol;Acc:HGNC:16673] | 9568 | -0.280 | -0.3160 | Yes |
| 52 | MTMR12 | myotubularin related protein 12 [Source:HGNC Symbol;Acc:HGNC:18191] | 9648 | -0.287 | -0.3104 | Yes |
| 53 | PPP1R14D | protein phosphatase 1 regulatory inhibitor subunit 14D [Source:HGNC Symbol;Acc:HGNC:14953] | 9650 | -0.287 | -0.2987 | Yes |
| 54 | PPP4R3A | protein phosphatase 4 regulatory subunit 3A [Source:HGNC Symbol;Acc:HGNC:20219] | 9676 | -0.289 | -0.2888 | Yes |
| 55 | PPP1R37 | protein phosphatase 1 regulatory subunit 37 [Source:HGNC Symbol;Acc:HGNC:27607] | 9858 | -0.307 | -0.2904 | Yes |
| 56 | CALM1 | calmodulin 1 [Source:HGNC Symbol;Acc:HGNC:1442] | 10136 | -0.334 | -0.2983 | Yes |
| 57 | DMPK | DM1 protein kinase [Source:HGNC Symbol;Acc:HGNC:2933] | 10331 | -0.354 | -0.2990 | Yes |
| 58 | PPP2R5E | protein phosphatase 2 regulatory subunit B'epsilon [Source:HGNC Symbol;Acc:HGNC:9313] | 10392 | -0.360 | -0.2889 | Yes |
| 59 | PPP2R1A | protein phosphatase 2 scaffold subunit Aalpha [Source:HGNC Symbol;Acc:HGNC:9302] | 10395 | -0.360 | -0.2743 | Yes |
| 60 | SBF1 | SET binding factor 1 [Source:HGNC Symbol;Acc:HGNC:10542] | 10406 | -0.361 | -0.2603 | Yes |
| 61 | PPP2R1B | protein phosphatase 2 scaffold subunit Abeta [Source:HGNC Symbol;Acc:HGNC:9303] | 10578 | -0.382 | -0.2581 | Yes |
| 62 | CALM2 | calmodulin 2 [Source:HGNC Symbol;Acc:HGNC:1445] | 10579 | -0.382 | -0.2425 | Yes |
| 63 | SAG | S-antigen visual arrestin [Source:HGNC Symbol;Acc:HGNC:10521] | 10655 | -0.388 | -0.2324 | Yes |
| 64 | BMP2 | bone morphogenetic protein 2 [Source:HGNC Symbol;Acc:HGNC:1069] | 10919 | -0.420 | -0.2357 | Yes |
| 65 | PHACTR2 | phosphatase and actin regulator 2 [Source:HGNC Symbol;Acc:HGNC:20956] | 11067 | -0.440 | -0.2292 | Yes |
| 66 | BMPR2 | bone morphogenetic protein receptor type 2 [Source:HGNC Symbol;Acc:HGNC:1078] | 11174 | -0.454 | -0.2189 | Yes |
| 67 | PPP1R12A | protein phosphatase 1 regulatory subunit 12A [Source:HGNC Symbol;Acc:HGNC:7618] | 11259 | -0.465 | -0.2064 | Yes |
| 68 | SHOC2 | SHOC2 leucine rich repeat scaffold protein [Source:HGNC Symbol;Acc:HGNC:15454] | 11440 | -0.494 | -0.2002 | Yes |
| 69 | RCAN3 | RCAN family member 3 [Source:HGNC Symbol;Acc:HGNC:3042] | 11455 | -0.496 | -0.1810 | Yes |
| 70 | ZEB2 | zinc finger E-box binding homeobox 2 [Source:HGNC Symbol;Acc:HGNC:14881] | 11644 | -0.524 | -0.1742 | Yes |
| 71 | PPP6R1 | protein phosphatase 6 regulatory subunit 1 [Source:HGNC Symbol;Acc:HGNC:29195] | 11739 | -0.539 | -0.1595 | Yes |
| 72 | LMTK2 | lemur tyrosine kinase 2 [Source:HGNC Symbol;Acc:HGNC:17880] | 11764 | -0.544 | -0.1391 | Yes |
| 73 | TESC | tescalcin [Source:HGNC Symbol;Acc:HGNC:26065] | 11793 | -0.548 | -0.1189 | Yes |
| 74 | SET | SET nuclear proto-oncogene [Source:HGNC Symbol;Acc:HGNC:10760] | 12000 | -0.593 | -0.1107 | Yes |
| 75 | ANKLE2 | ankyrin repeat and LEM domain containing 2 [Source:HGNC Symbol;Acc:HGNC:29101] | 12043 | -0.604 | -0.0893 | Yes |
| 76 | PPP1R15A | protein phosphatase 1 regulatory subunit 15A [Source:HGNC Symbol;Acc:HGNC:14375] | 12107 | -0.622 | -0.0687 | Yes |
| 77 | PPP6R3 | protein phosphatase 6 regulatory subunit 3 [Source:HGNC Symbol;Acc:HGNC:1173] | 12224 | -0.652 | -0.0511 | Yes |
| 78 | PPP1R10 | protein phosphatase 1 regulatory subunit 10 [Source:HGNC Symbol;Acc:HGNC:9284] | 12568 | -0.780 | -0.0459 | Yes |
| 79 | PPP3R1 | "protein phosphatase 3 regulatory subunit B, alpha [Source:HGNC Symbol;Acc:HGNC:9317]" | 12653 | -0.829 | -0.0186 | Yes |
| 80 | PHACTR1 | phosphatase and actin regulator 1 [Source:HGNC Symbol;Acc:HGNC:20990] | 12760 | -0.923 | 0.0109 | Yes |
Table: GSEA details [plain text format]

  

Fig 2: GOMF\_PHOSPHATASE\_REGULATOR\_ACTIVITY      
 Blue-Pink O' Gram in the Space of the Analyzed GeneSet

  

Fig 3: GOMF\_PHOSPHATASE\_REGULATOR\_ACTIVITY: Random ES distribution      
 Gene set null distribution of ES for **GOMF\_PHOSPHATASE\_REGULATOR\_ACTIVITY**
